# Enhanced therapeutic effect of Adriamycin on multidrug resistant breast cancer by the ABCG2-siRNA loaded polymeric nanoparticles assisted with ultrasound

**DOI:** 10.18632/oncotarget.6085

**Published:** 2015-11-09

**Authors:** Min Bai, Ming Shen, Yanwei Teng, Ying Sun, Fan Li, Xiangyu Zhang, Yuanyuan Xu, Yourong Duan, Lianfang Du

**Affiliations:** ^1^ Department of Ultrasound, Shanghai First People's Hospital, School of Medicine, Shanghai Jiao Tong University, Shanghai 200080, People's Republic of China; ^2^ State Key Laboratory of Oncogenes and Related Genes, Shanghai Cancer Institute, Renji Hospital, School of Medicine, Shanghai Jiao Tong University, Shanghai 200032, People's Republic of China

**Keywords:** siRNA delivery, UTMD, gene silencing, combination therapy, xenograft nude mice model

## Abstract

The overexpression of the breast cancer resistance protein (ABCG2) confers resistance to Adriamycin (ADR) in breast cancer. The silencing of ABCG2 using small interfering RNA (siRNA) could be a promising approach to overcome multidrug resistance (MDR) in cancer cells. To deliver ABCG2-siRNA effectively into breast cancer cells, we used mPEG-PLGA-PLL (PEAL) nanoparticles (NPs) with ultrasound-targeted microbubble destruction (UTMD). PEAL NPs were prepared with an emulsion-solvent evaporation method. The NPs size was about 131.5 ± 6.5 nm. The siRNA stability in serum was enhanced. The intracellular ADR concentration increased after the introduction of siRNA-loaded NPs. After intravenous injection of PEAL NPs in tumor-bearing mice, the ABCG2-siRNA-loaded NPs with UTMD efficiently silenced the ABCG2 gene and enhanced the ADR susceptibility of MCF-7/ADR (ADR resistant human breast cancer cells). The siRNA-loaded NPs with UTMD + ADR showed better tumor inhibition effect and good safety *in vivo*. These results indicate that ADR-chemotherapy in combination with ABCG2-siRNA is an attractive strategy to treat breast cancer.

## INTRODUCTION

Chemotherapy is very important in the comprehensive treatment of breast cancer and has been suggested to be a major treatment to avoid the recurrence of cancer after surgery [1]. However, tumors might be intrinsically resistant to chemotherapy or acquire multidrug resistance (MDR) during treatment [2–4]. A well-established cause of MDR involves increased drug efflux from cancer cells mediated through members of the ATP-binding cassette (ABC) transporter family [5, 6]. Among ABC transporter family members, a novel protein, the breast cancer resistance protein (ABCG2), has been detected in a variety of malignancy tumors, such as breast cancer [7, 8]. Experimental evidence suggests that ABCG2 overexpression is associated with reduced intracellular drug concentration and the decreased cytotoxicity of numerous anticancer agents, including anthracyclines and camptothecins, by enhancing drug efflux [9, 10]. Therefore, knocking down ABCG2 gene expression might effectively reverse drug resistance in breast cancer cells.

RNA interference (RNAi), an emerging gene knockdown technology, is more specific and efficient compared with traditional gene knockdown techniques [11, 12]. RNAi is of 21 to 23 nt small interfering RNAs (siRNA) that facilitate the silencing of complementary target mRNA, thereby silencing gene expression [13–15]. However, the delivery of siRNA remains a major hurdle in the full exploitation of siRNA-based therapeutics. It is difficult for siRNA to pass through the cell membrane, as both of these entities are negatively charged [16, 17]. Therefore, safe and effective approaches for the targeting *in vivo* delivery of these molecules are appealing [18].

Monomethoxy polyethylene glycol-polylactic acid/glycolic acid-poly-L-lysine triblock copolymer (mPEG-PLGA-PLL, PEAL) is a cationic polymer with blocks to get nanoparticles with hydrophilic shell and hydrophobic core, suitable for carrying various genes [19–21]. The protonated amino group of PLL could bind with negatively charged siRNA to increase the entrapment efficiency [22]. The directional rupture of microbubbles (MB) by low-frequency ultrasound (UTMD) facilitates the penetration of PEAL NPs into the cell membrane and subsequent into cells [23–25]. In the present study, we used PEAL NPs as siRNA deliver system assistant with UTMD to treat breast cancer.

To investigate the effects of ABCG2-siRNA in reversing MDR in mice bearing Adriamycin-resistant human breast cancer cells (MCF-7/ADR) xenograft tumors, using Adriamycin-sensitive human breast cancer cells (MCF-7/S) xenograft tumors as a control, we prepared siRNA-loaded PEAL NPs by the emulsion-solvent evaporation method for systemic siRNA delivery (Supplementary Scheme S1) [26]. The resensitization effects of breast cancer towards ADR after ABCG2-siRNA transfection and *in vivo* safety of siRNA-loaded PEAL NPs were also investigated.

## RESULTS

### Preparation and characterization of NPs

**Figure 1:**
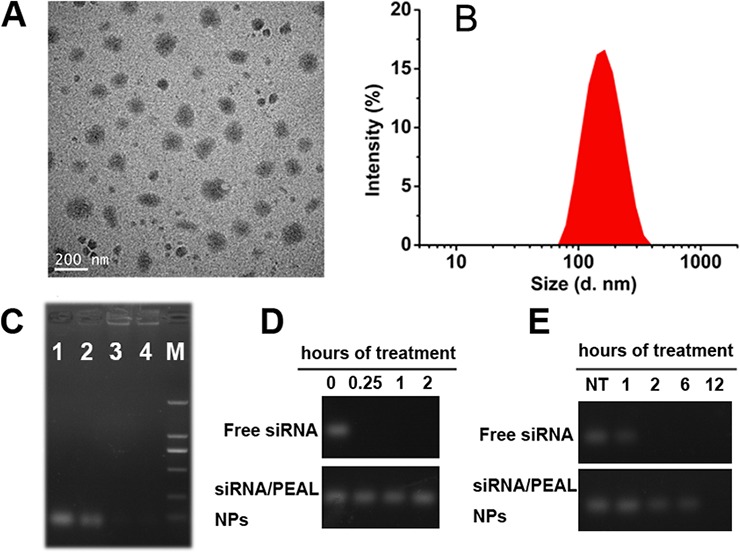
Characterization of siRNA-loaded PEAL NPs **A.** TEM image; **B.** Particle size and size distribution; **C.** The loading capacity of PEAL to siRNA (1: Naked siRNA, 2: NP_2.5_, 3: NP_1.0_, 4: NP_0.5_); **D.** Anti-enzymatic activity; E. The serum stability.

The hydrodynamic diameter of siRNA-loaded PEAL NPs, measured using dynamic light scattering (DLS), was 131.5 ± 6.5 nm with a PDI of 0.162 (Figure 1B). Transmission electron microscopy (TEM) revealed that siRNA-loaded PEAL NPs were spherical particles and dispersed well (Figure 1A). Gel retardation assay confirmed that siRNA could be completely packaged into PEAL NPs when the content of siRNA in feed was no more than 1 nmol (Figure 1C). The contents of intact siRNA in various formulations after treatment with 1mU of RNase or 50% serum solution for different time were shown in Figure 1D and Figure 1E, respectively. According to Figure 1E, free siRNA degraded after exposed to RNase within 2 h. In contrast, almost no degradation was observed in siRNA loaded PEAL NPs within 2 h, demonstrating that PEAL NPs protected siRNA from enzymatic degradation in serum.

### Increased drug sensitivity in siRNA-transfected cells

The IC_50_ values of MCF-7/S and MCF-7/ADR cells treated with different transfection agents are shown in Table 1, demonstrating that 0.558 μg/mL of ADR induced 50% reduction in MCF-7/S cell proliferation. However, for MCF-7/ADR cells, the necessary concentration for 50% inhibition of cell proliferation was 242 μg/mL with a resistance index (R) of nearly 440. As expected, the exposure of MCF-7/ADR cells to ABCG2-specific siRNAs prior to the addition of ADR significantly decreased the IC_50_ of ADR (IC_50_ values of 144, 124, 97 and 76 μg/mL for free siRNA, free siRNA with UTMD, siRNA-loaded PEAL NPs, and siRNA-loaded PEAL NPs with UTMD treatments, respectively). In the presence of UTMD, the IC_50_ values were reduced in all groups. According to the fold-reversal (FR) values of resistant cells (Table 1), siRNA-loaded PEAL NPs with UTMD induced the 3-fold resensitization of MCF-7/ADR cells to ADR.

**Table 1:** IC_50_ values of ADR in MCF-7/S and MCF-7/ADR cells after transfection with different siRNA formulations

Cells	Groups	IC_50_ (μg/mL)	R	FR
MCF-7/S	ADR	0.558±0.015	-	-
	ADR	242±9	434	-
	ADR+ Free siRNA	144±10*	258	1.68
MCF-7/ADR	ADR+ Free siRNA +UTMD	124±7*^	240	1.81
	ADR+ siRNA loaded PEAL NPs	97±9*	174	2.49
	ADR+ siRNA loaded PEAL NPs +UTMD	76±11*^	136	3.18

The * stands for significant difference contrasted with ADR group (MCF-7/ADR cells). The ^ stands for significant difference contrasted with the corresponding group just without UTMD.

### Increased drug accumulation in siRNA-transfected cells

**Figure 2:**
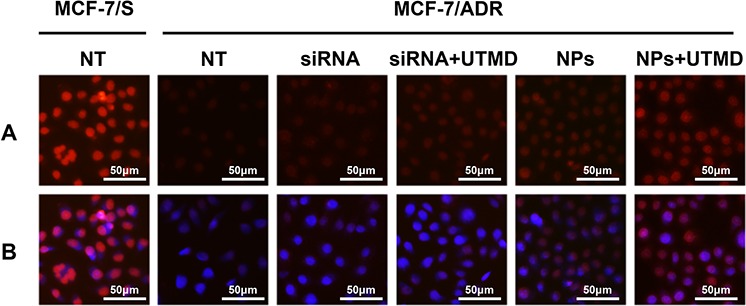
The impacts of ABCG2 knockdown through different siRNA formulations on intracellular drug accumulation

To conduct a more intuitive examination for the role of ABCG2-siRNA in reducing drug efflux, we observed the intracellular concentration of ADR in untreated and siRNA-transfected cells. The results showed that the transfection of siRNAs into MCF-7/ADR cells enhanced intracellular drug accumulation (Figure 2). A more substantial increase was observed in ABCG2-siRNA-loaded PEAL NPs transfected MCF-7/ADR cells, compared with non-transfected or free ABCG2-siRNA transfected cells. And as expected, the cells transfected with siRNA in the presence of UTMD showed more intense drug accumulation than those without UTMD.

### *In vivo* tumor growth inhibition

**Figure 3:**
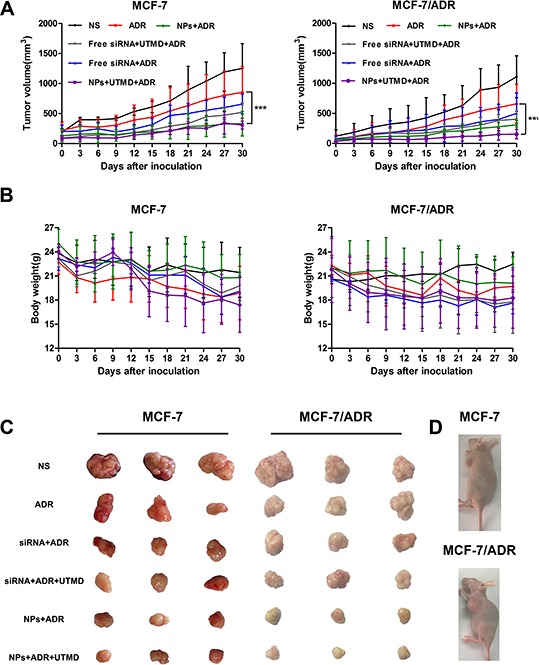
**A.** Inhibition of tumor growth in MCF-7/S and MCF-7/ADR subcutaneous xenograft tumors after the intravenous injection of various siRNA formulations. **B.** The changes in the body weight of mice bearing MCF-7/S and MCF-7/ADR subcutaneous xenograft tumors over the treatment period. **C.** Images of MCF-7/S and MCF-7/ADR subcutaneous xenograft tumors at the treatment endpoint. **D.** Representative pictures of tumor-bearing mice after treatment for 20 days.

To investigate the tumor therapeutic efficiency of systemic siRNA delivery *in vivo*, nude mice bearing MCF-7/S or MCF-7/ADR tumor xenografts were subjected to various formulations at 1.3 mg/kg ABCG2-siRNA and 5 mg/kg ADR every 3 days. As shown in Figure 3A, non-ignorable effects on tumor growth were observed after treatment with ADR alone compared with the (normal saline) NS group. Both siRNA-loaded PEAL NPs alone and in the presence of UTMD showed significantly increased inhibition on tumor growth. As expected, siRNA-loaded PEAL NPs + UTMD exhibited stronger tumor growth inhibition compared with siRNA-loaded PEAL NPs alone, particularly for mice bearing MCF-7/ADR tumor xenografts.

After final treatment, all tumors were collected and photographed (Figure 3C). The results revealed that ADR in combination with siRNA mediated by PEAL NPs + UTMD could effectively inhibit tumor growth, and these results were consistent with the results of the tumor volume assay.

With respect to safety evaluation (Figure 3B), compared with the NS group, the mice in the treatment group showed varying degrees of decreased body weights during the entire experimental period, likely resulting from the toxicity of ADR.

**Figure 4:**
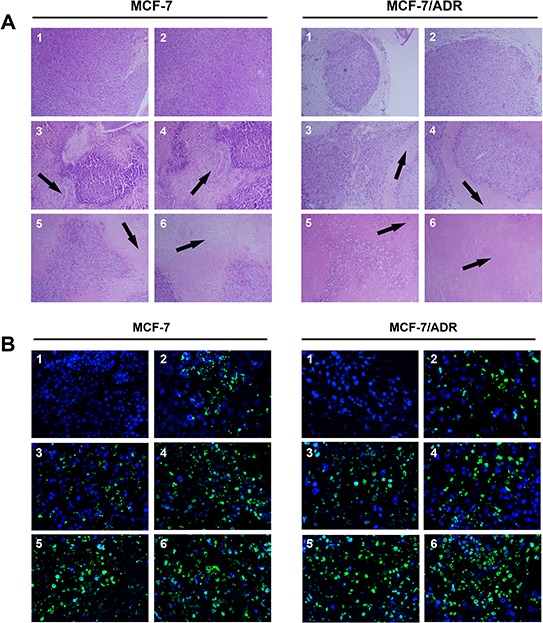
H & E staining (A. × 100) and TUNEL analysis (B. × 400) of tumor tissues after treatment with various siRNA formulations The tumor tissues were collected at 24 h after the last injection. (1) NC control, (2) ADR, (3) Free siRNA + ADR, (4) Free siRNA + UTMD + ADR, (5) SiRNA-loaded PEAL NPs + ADR, (6) SiRNA-loaded PEAL NPs + UTMD + ADR.

Figure 4 shows the results of the histological analysis and TUNEL staining of tumor tissues after treatment. Compared with the NS group, siRNA-loaded PEAL NPs + UTMD markedly decreased the number of cancer cells, indicating that the administration of siRNA-loaded PEAL NPs + UTMD increased sensitivity to ADR and effectively enhanced cell apoptosis, leading to considerable antitumor efficacy.

### *In vivo* gene silencing efficiency

**Figure 5:**
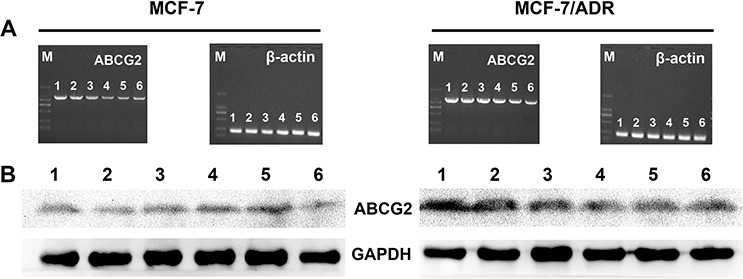
*In vivo* gene silencing efficiency of different siRNA formulations on MCF-7/S and MCF-7/ADR xenograft tumors analyzed at gene A. and protein B. levels (1) NS control, (2) ADR, (3) Free siRNA + ADR, (4) Free siRNA + UTMD + ADR, (5) SiRNA-loaded PEAL NPs + ADR, (6) SiRNA-loaded PEAL NPs + UTMD + ADR.

The *in vivo* gene silencing of ABCG2-siRNA-loaded PEAL NPs was evaluated in animals bearing MCF-7/S and MCF-7/ADR tumor xenografts. To demonstrate that the retarded tumor growth observed after treatment with siRNA-loaded PEAL NPs + UTMD plus ADR was associated with ABCG2 downregulation in tumor cells, the mice were executed after treatment for 30 days, and all tumors were collected to extract total mRNA or protein to assess the levels of ABCG2 mRNA and protein expression using RT-PCR and WB analyses, respectively. As shown in Figure 5, ABCG2-siRNA-loaded PEAL NPs + UTMD significantly suppressed the expression of the target gene in tumor xenografts, consistent with the gene silencing effects observed *in vitro*.

### Systemic toxicity evaluation

To evaluate the safety of siRNA-loaded PEAL NPs assisted ADR *in vivo*, the physiological states of mice were recorded daily during injection. Weight loss was observed during the experimental period in ADR group.

**Figure 6:**
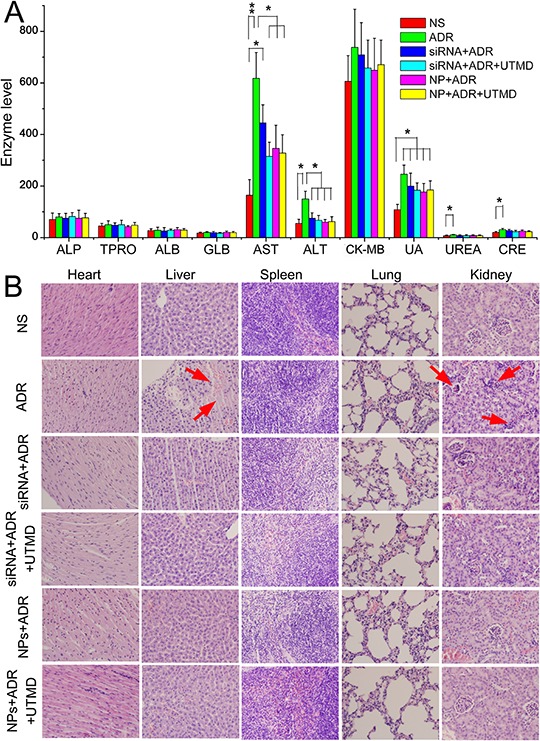
Safety result A. Indices of hepatic, heart and renal function measured at 24 hours after the final injection of different siRNA formulations in nude mice **B.** Histopathology of H & E-stained major organs from mice after treatment with PBS and different siRNA formulations. ALP: alkaline phosphatase, TPRO: total protein, ALB: albumin, GLB: globulin, AST: aspartate transaminase, ALT: alanine transaminase, CK-MB: creatine phosphate kinase isoforms - MB, UA: uric acid, UREA: urea, CRE: creatinine. The * stands for significant difference (*P* < 0.05) and ** stand for *P* < 0.01. The pathological changes were marked with red arrows.

The blood was collected on the last day of the experiment to determine a part of hepatic, heart and renal function indices. As shown in Figure 6A, the aspartate transaminase (AST) in ADR group was higher than that in NS group (*P* < 0.01). The level in siRNA+ADR group was lower than in the ADR group, but still higher than the NS group (*P* < 0.05). However, the AST in the other siRNA groups were lower than in ADR group (*P* < 0.05) and no significant difference than in NS group. Similar trend was observed in alanine transaminase (ALT) level except the level in siRNA+ADR group, like in the other siRNA groups, was no diference with in the NS group. There was no significant difference in creatine phosphate kinase isoforms - MB (CK-MB) level as an indicator of cardiac function. The uric acid (UA) level in ADR group was higher than in NS group (*P* < 0.05). The levels in the siRNA groups were all lower than in ADR group but without significant difference. Similar trend were in urea (UREA) and creatinine (CRE) levels.

Histopathological photos were showed in Figure 6B. Ecrosis in liver and glomerular shrinkage were observed in ADR group. There were no obvious pathological changes was observed in other groups.

The toxicity result showed that the ADR at 5mg/kg was harmful to liver and kidney. The siRNA preparations reduced the toxicity of ADR to a certain extent.

## DISCUSSION

ABCG2, the second member of the subfamily G of the human ABC transporter superfamily, has been detected in a large number of hematological malignancies and solid tumors, indicating that this transporter might play an important role in the MDR of cancers [10]. Transfection studies from different laboratories confirmed that the overexpression of ABCG2 in different cell types confers resistance to various anticancer agents and reduces drug accumulation in the cell [9, 29, 30].

Many studies have been reported down-regulated ABCG2 expression and reverse drug resistance in breast cancer. Sridhar K *et al*. reported that fumitremorgin C could reverse the MDR in cells transfected with the ABCG2 gene [31]. However, the neurotoxicity of this toxin, characterized by tremors and tetanic convulsions in mice, prevented the use of this chemical in *in vivo* experiments [32]. Yasuo Imai *et al*. reported that estrone and 17β-estradiol could reverse ABCG2-mediated MDR [33]. However, the coadministration or intake of these drugs with ABCG2-substrate antitumor agents might result in the alteration of the pharmacokinetics of these compounds and increase the toxicity of the antitumor drugs in the recipient patients [34]. With increasing understanding of the mechanisms of RNAi, siRNA has been proposed as an effective approach to specifically silence ABCG2 in cancer cells. MCF-7/ADR cells overexpress ABCG2. Therefore, we selected MCF-7/ADR as model cells, and MCF-7/S cells were used as a control. Here, we have designed and synthesized ABCG2-siRNA to specifically and effectively down-regulate breast cancer resistance-related protein (BCRP) expression in tumor cells *in vitro*. A major obstacle to the clinical application of RNAi is the disadvantageous pharmacokinetic profile, which strongly hinders this molecule from achieving effective concentrations at target sites *in vivo*. Therefore, delivery strategies that stabilize siRNA and enhance intracellular uptake *in vivo* are needed.

The BCRP is highly expressed on the apical membranes of the placental syncytiotrophoblasts, the intestinal epithelium, the liver hepatocytes, the endothelial cells of brain microvessels, and the renal proximal tubular cells, contributing to the absorption, distribution, and elimination of drugs and endogenous compounds as well as tissue protection against xenobiotic exposure [35]. This may be the reason that the miRNA preparations showed protecting effect to liver and kidney in the safety evaluation (Figure 6).

In a previous study, we reported the synthesis of a new polymer, PEAL, with excellent biocompatibility *in vitro* and high encapsulation efficiency for the stable delivery of siRNA [36]. In the present study, we prepared the ABCG2-siRNA-loaded PEAL NPs using the emulsification-solvent evaporation method. The resulting NPs were uniform in size, which would be suitable for passive tumor targeting of drug delivery through the EPR effect [37, 38]. The outer layer of NPs was covered with hydrophilic mPEG, which improve the stability and biocompatibility of this nanoparticle [39, 40]. Also it can enhance the mean residence time (MRT) of NPs in blood cycle to get more opportunity to tumor [41]. Apparently, these nanoparticles protected siRNA from serum-driven nuclease digestion efficiently. The siRNAs, delivered by NPs, effectively knocked down ABCG2 gene expression and reduced drug efflux, thereby increasing the sensitivity of resistant cells to ADR. The NPs led to specifically silences genes without inducing obvious organ toxicity (Figure 6), suggesting the potential clinical application.

UTMD was observed Ultrasound induced microbubble cavitation can cause enhanced permeability across natural barriers of tumors such as vessel walls or cellular membranes, allowing for enhanced therapeutic delivery into the target tissues. Titanium dioxide nanoparticles was reported used for tumor image. The optical signal intensity suggests that light penetration depth is significantly enlarged by ultrasound [42]. Ultrasound was reported to enhance PLGA-PEG-NP effect. As the pressures increased from 1.7 to 6.9 MPa, the amount of nanoparticles deposited in cancer xenografts was increased from 4- to 14-fold, and the median penetration depth of extravasated nanoparticles was increased from 1.3-fold to 3-fold, compared to control conditions without ultrasound [43]. Ultrasound enhanced drug distribution in tumor may by the way of inducing extravasation, improving penetration through the extracellular matrix and enhancing cell uptake [44].

Taken together, these results demonstrated that ABCG2-siRNA-loaded PEAL NPs are promising tools to inhibit ABCG2 overexpression in MCF-7/ADR cells and sensitize its response to drugs. Some insight researches, such as ADR pharmacodynamics affected by miRNA NPs will be carried out in future work.

## MATERIALS AND METHODS

### Materials

MPEG-PLGA-PLL was synthesized in our laboratory. The molecular weight of PLGA (LA:GA 7:3, molar ratio) was about 7000 and that of mPEG was 2000. The total molecular weight (Mw) of the polymer was about 10,000. ABCG2-siRNA were designed and synthesized by Ruibo Biotechnology Inc. (Guangzhou, China) (Table 2). Doxorubicin hydrochloride (Adriamycin, ADR) was purchased from Beijing Huafenglianbo Technology Co., Ltd. TRIzol^®^ reagent was purchased from Invitrogen (Gibco, Paisley, Renfrewshire, Scotland, UK), and 3-(4,5-dimethylthiazol-2-yl)-2,5-diphenyltetrazolium bromide (MTT) was purchased from Qianchen Biotechnology Inc. (Shanghai, China). SonoVue^®^, diluted to 5 ml with saline to form microbubbles, was purchased from Bracco Diagnostics Inc. (Geneva, Switzerland).

**Table 2:** ABCG2-siRNA and primers used in the study

Name, accession no.	Sequence (5′-3′)
ABCG2, NM_004827	Sense: 5′-TCAAACCTGGTCTCAACGCC-3′ Antisense: 5′-CGGTAGTATCCGCTGATGTATTC-3′
β-actin, NM_001101	Sense: 5′-CAGAGCAAGAGAGGCATCCT-3′ Antisense: 5′-GTTGAAGGTCTCAAACATGATC-3′
ABCG2-siRNA	Sense: 5′-CUGGAGAUGUUCUGAUAAA dTdT-3′ Antisense: 3′-dTdT GACCUCUACAAGACUAUUU-5′

### Cell culture

MCF-7/S cells were purchased from the Cell Bank of the Chinese Academy of Sciences (Shanghai, China), and the multidrug-resistant variant MCF-7/ADR was established and maintained in our laboratory. All cells were cultured in RPMI-1640 medium (Biosera, UK) supplemented with 10% fetal bovine serum (Gibco), and 1% penicillin and streptomycin (Biosera). MCF-7/ADR cells were grown in the RPMI 1640 complete medium with 1 μg/mL ADR to maintain drug-resistance. The cells were cultured at 37°C in a humidified atmosphere containing 5% CO_2_.

### Experimental facilities of ultrasound

The therapeutic ultrasound apparatus Topteam 161 (Chattanooga Company, CA, USA), with a frequency of 1 MHz, pulse repetition frequency of 100 Hz and 25 mm^2^ cross sectional probe area, was used to enhance siRNA delivery. Prior to insonation, the ultrasound probe was placed on the bottom of the culture plate with a small amount of couplants on the surface of the probe to form a conductive pathway of ultrasound waves from transducer to cells.

### Western blot

Total protein was extracted using lysis buffer supplemented with 1% PMSF, and the concentration was determined using a BCA protein assay kit (Sigma, St Louis, MO, USA). Approximately 20 μg of total protein from each sample was loaded onto 10% SDS-polyacrylamide gels and subsequently transferred to nitrocellulose membranes (Millipore, Massachusetts, USA). The membranes were incubated with rabbit antibody against ABCG2 (1:300) at 4°C overnight. Glyceraldehyde-3-phosphate dehydrogenase (GAPDH) was used as an internal standard to normalize protein expression, and the membranes were incubated with GAPDH monoclonal antibody (1:2500) simultaneously. The membranes were subsequently washed with TBST (Tris-HCl+Tween) and incubated with HRP-conjugated secondary goat anti-rabbit or anti-mouse antibody (1:2000) at 37°C for 1 h. The protein-antibody complexes were analyzed using chemiluminescence. All antibodies used in this study were purchased from Tuoran Biotechnology Inc. (Shanghai, China).

### Reverse transcription PCR (RT-PCR)

The transfected cells were harvested, and total RNA was extracted using TRIzol^®^ reagent, according to the manufacturer's instructions. Approximately 5 μg of total RNA from each sample was converted to cDNA using a commercially available RT-PCR kit (TaKaRa, Kyoto, Japan). The primers used are shown in Table 2. The PCR conditions were enzyme activation at 95°C for 3 min, followed by 34 cycles of 95°C for 30 sec, 55°C for 30 sec, and 72°C for 110 sec, with a final extension cycle at 72°C for 5 min. The PCR products were analyzed through electrophoresis on 1% agarose gels containing 1 μg/mL ethidium bromide. The gels were visualized using an EC3 Imaging System (UVP, LLC, Upland, CA, USA).

### Preparation and characterization of NPs

ABCG2-siRNA-loaded PEAL NPs were prepared by double emulsified method. Briefly, 2 mg PEAL in dichloromethane (1 mg/mL) was emulsified in a solution containing 1 nmol of siRNA in diethyl pyrocarbonate (DEPC)-treated water by sonication for 50 s in an ice bath. The primary emulsion obtained was further emulsified in 2 mL Pluronic 188 solution (1.0%, w/v) by sonication to form a water-in-oil-in-water emulsion. The dichloromethane was subsequently removed using a rotary evaporator (Taikang Biotechnology Inc., Shaanxi, China). The siRNA-loaded NPs were denoted as NP_1.0_, where the subscript represents the number of the siRNA molecule as nmol.

The particle size and polydispersity index (PDI) of the NPs were measured at 25°C using a Zetasizer IV analyzer (Malvern Zetasizer Nano ZS90, UK). The morphology was observed through transmission electron microscopy (TEM, JEOL, Tokyo, Japan). To assess the integrity of the siRNAs after entrapment in PEAL NPs, a gel retardation assay was performed.

### Gel retardation assay

Gel retardation electrophoresis was used to detect the loading capacity of PEAL to siRNA, as well as anti-enzymatic activity and serum stability of the siRNA molecules after entrapment in PEAL NPs. Samples of naked siRNA and siRNA-loaded PEAL NPs, containing different siRNA contents (siRNA concentration was fixed at 100 nM), were respectively mixed with serum at a ratio of 1:1 (v/v) or 1 mU of RNase, and the resulting mixtures were incubated at 37°C. At different time points, aliquots of each sample were collected for analysis. All the samples were loaded onto 1% agarose gels containing 1 μg/mL ethidium bromide.

### Cell proliferation assay

MCF-7/S and MCF-7/ADR cells were seeded onto 96-well plates at a density of 5 × 10^3^ cells/well, incubated overnight and subsequently transfected with different siRNA and UTMD treatment. After transfection for 48 h, the culture medium was removed, followed by the addition of ADR at a series of concentrations. The cells were incubated for 48 h under normal maintenance conditions, and cell viability was measured using an MTT assay. The absorbance was measured at 570 nm using a microplate reader (Bio-Rad Laboratories Inc., Hercules, CA, USA). The cell viability was normalized to that of cells treated with phosphate buffered saline (PBS). The resistance index (R) and fold-reversal (FR) values were calculated using the following equations [27, 28]:

$$\begin{array}{l} \text{R}={\text{IC}}_{\text{50 ADR, MCF-7/ADR}}{\text{/IC}}_{\text{50 ADR, MCF-7/S}}\text{;}\\ \text{FR}={\text{IC}}_{\text{50 ADR, MCF-7/ADR}}{\text{/IC}}_{\text{50 ADR+siRNA, MCF-7/ADR}}\text{.} \end{array}$$

### Intracellular drug accumulation

The effect of ABCG2-siRNA on cellular ADR accumulation was determined using an inverted fluorescence microscope (Huarui Chemical Instrument Inc., Guangdong, China). Briefly, 1 × 10^5^ MCF-7/ADR cells were seeded onto 6-well plates and subsequently transfected by siRNA preparations in the absence or presence of UTMD. The final concentration of siRNA was 100 nM. After 48 h, the cells were incubated with 1 μg/mL ADR for 1 h at 37°C. Subsequently, the cells were washed three times with PBS, fixed with 4% paraformaldehyde and the nuclei were stained with DAPI (4′,6-diamidino-2-phenylindole staining solution). Subsequently, the cells were subjected to fluorescence analysis to determine the intracellular ADR accumulation.

### *In vivo* tumor growth inhibition study

Female BALB/c nude mice, weighing 15–18 g, were purchased from Shanghai Slac Laboratory Animal Co., Ltd. (Shanghai, China). The xenograft tumor model was generated through the subcutaneous injection of MCF-7/S or MCF-7/ADR cells (5 × 10^6^ for each mouse) in the flank regions of the mice. When the tumor volume was approximately 100 mm^3^, the mice were randomly divided into 6 groups and treated with different therapy: (1) 0.9% normal saline (NS); (2) NS + ADR; (3) free siRNA + ADR; (4) free siRNA + UTMD + ADR; (5) siRNA-loaded PEAL NPs + ADR; and (6) siRNA-loaded PEAL NPs + UTMD + ADR. NS, siRNA and siRNA-loaded PEAL NPs were through intravenous injection. ADR was through intraperitoneal injection [45]. The body weight and tumor size of each mouse were measured every 3 days. The diameter of the tumors was measured using a vernier caliper. The estimated tumor volume was calculated using the formula: volume= 0.5 × length × width^2^. At 24 h after the final injection, all tumors were collected for further analysis.

### *In vivo* gene silencing efficiency

For ABCG2 protein measurements, the tumors were cut into pieces and subsequently homogenized in lysis buffer supplemented with 1% PMSF (phenylmethanesulfonyl fluoride). After centrifuged at 12,000 rpm for 5 min at 4°C, the supernatant was collected for further analysis.

The expression of ABCG2 mRNA in the tumors was analyzed through RT-PCR. The tumor tissue (~50 mg) was homogenized in 1 mL of pre-cooling TRIzol reagent and subsequently incubated at room temperature for 5 min to facilitate the complete separation of protein-nucleic acid complexes. After centrifugation at 12,000 rpm for 5 min at 4°C, the supernatant was collected and analyzed through RT-PCR as described above.

### H&E staining and TUNEL analysis

To conduct histological analysis, the tumors were fixed in 4% paraformaldehyde for 24 h. After deparaffinization, the tissue sections (5 mm) were stained with hematoxylin/eosin (H&E). The tumor cell apoptosis was determined using the One-step TdT-mediated dUTP Nick-End Labeling (TUNEL) Apoptosis Kit according to the manufacturer's instructions (Beyotime, China). All sections were examined using an inverted fluorescence microscope. TUNEL-positive cells were shown in green.

### Safety evaluation

The mice were treated respectively with 0.9% NS (control), ADR, free siRNA, free siRNA + UTMD, siRNA-loaded PEAL NPs or siRNA-loaded PEAL NPs + UTMD once every 3 days for 5 times. The siRNA dose was 1.3 mg/kg. The ADR dose was 5 mg/kg. At 24^th^ hour after the final injection, serum samples were collected and measured to assess hepatic and renal damage. After the mice were sacrificed, the major organs, such as liver, heart, spleen, lung and kidney of each mouse were collected, fixed, and processed for H&E staining to evaluate toxicity.

### Statistical Analysis

All data were performed in triplicate and shown as the means ± standard deviation (SD). Student's *t*-test was used to compare two groups of data. GraphPad Prism (GraphPad Software Inc., California) was used to obtain graphs and statistics. Significant values were designated as *p < 0.05, **p < 0.01, and ***p < 0.001.

## SUPPLEMENTARY SCHEME FIGURE



## References

[1] Early Breast Cancer Trialists' Collaborative Group (2005). Effects of chemotherapy and hormonal therapy for early breast cancer on recurrence and 15-year survival: an overview of the randomised trials. Lancet.

[2] Gottesman MM (2002). Mechanisms of cancer drug resistance. Annual Review of Medicine.

[3] Longley D, Johnston P (2005). Molecular mechanisms of drug resistance. American Journal of Pathology.

[4] Ling V (1997). Multidrug resistance: molecular mechanisms and clinical relevance. Cancer Chemotherapy and Pharmacology.

[5] Glavinas H, Krajcsi P, Cserepes J, Sarkadi B (2004). The role of ABC transporters in drug resistance, metabolism and toxicity. Current Drug Delivery.

[6] Schinkel AH, Jonker JW (2003). Mammalian drug efflux transporters of the ATP binding cassette (ABC) family: an overview. Advanced Drug Delivery Reviews.

[7] Staud F, Pavek P (2005). Breast cancer resistance protein (BCRP/ABCG2). International Journal of Biochemistry & Cell Biology.

[8] Zhou S, Schuetz JD, Bunting KD, Colapietro AM, Sampath J, Morris JJ, Lagutina I, Grosveld GC, Osawa M, Nakauchi H (2001). The ABC transporter Bcrp1/ABCG2 is expressed in a wide variety of stem cells and is a molecular determinant of the side-population phenotype. Nature Medicine.

[9] Doyle LA, Ross DD (2003). Multidrug resistance mediated by the breast cancer resistance protein BCRP (ABCG2). Oncogene.

[10] Mao Q, Unadkat JD (2005). Role of the breast cancer resistance protein (ABCG2) in drug transport. Aaps Journal.

[11] Ee PR, He X, Ross DD, Beck WT (2004). Modulation of breast cancer resistance protein (BCRP/ABCG2) gene expression using RNA interference. Molecular Cancer Therapeutics.

[12] Li W, Zhou G, Song X, Chi W, Ren R, Wang X (2004). Modulation of BCRP mediated atypical multidrug resistance phenotype by RNA interference. Neoplasma.

[13] Lares MR, Rossi JJ, Ouellet DL (2010). RNAi and small interfering RNAs in human disease therapeutic applications. Trends in Biotechnology.

[14] Reynolds A, Leake D, Boese Q, Scaringe S, Marshall WS, Khvorova A (2004). Rational siRNA design for RNA interference. Nature Biotechnology.

[15] Xia H, Mao Q, Paulson HL, Davidson BL (2002). siRNA-mediated gene silencing in vitro and in vivo. Nature Biotechnology.

[16] Wullner U, Neef I, Tur MK, Barth S (2009). Targeted delivery of short interfering RNAs-strategies for in vivo delivery. Recent Patents on Anti-Cancer Drug Discovery.

[17] Shim MS, Kwon YJ (2010). Efficient and targeted delivery of siRNA in vivo. Febs Journal.

[18] Blow N (2007). Small RNAs: delivering the future. Nature.

[19] Liu P, Yu H, Sun Y, Zhu M, Duan Y (2012). A mPEG-PLGA-b-PLL copolymer carrier for adriamycin and siRNA delivery. Biomaterials.

[20] Du J, Sun Y, Shi Q, Liu P, Zhu M, Wang C, Du LF, Duan Y (2012). Biodegradable nanoparticles of mPEG-PLGA-PLL triblock copolymers as novel non-viral vectors for improving siRNA delivery and gene silencing. International Journal of Molecular Sciences.

[21] Liu P, Sun Y, Wang Q, Sun Y, Li H, Duan Y (2014). Intracellular trafficking and cellular uptake mechanism of mPEG-PLGA-PLL and mPEG-PLGA-PLL-Gal nanoparticles for targeted delivery to hepatomas. Biomaterials.

[22] Walter F, Vicens Q, Westhof E (1999). Aminoglycoside(ndash)RNA interactions. Current Opinion in Chemical Biology.

[23] Bekeredjian R, Kuecherer HF, Kroll RD, Katus HA, Hardt SE (2007). Ultrasound-targeted microbubble destruction augments protein delivery into testes. Urology.

[24] Shen Z, Brayman A, Chen L, Miao C (2008). Ultrasound with microbubbles enhances gene expression of plasmid DNA in the liver via intraportal delivery. Gene Therapy.

[25] Bekeredjian R, Chen S, Pan W, Grayburn PA, Shohet RV (2004). Effects of ultrasound-targeted microbubble destruction on cardiac gene expression. Ultrasound in Medicine and Biology.

[26] Watts P, Davies M, Melia C (1989). Microencapsulation using emulsification/solvent evaporation: an overview of techniques and applications. Critical Reviews in Therapeutic Drug Carrier Systems.

[27] Iseri OD, Kars MD, Eroglu S, Gunduz U (2009). Drug resistant MCF-7 cell lines also developed cross-resistance to structurally unrelated anticancer agents. Uhod-Uluslararasi Hematoloji-Onkoloji Dergisi.

[28] Wu H, Hait WN, Yang JM (2003). Small interfering RNA-induced suppression of MDR1 (P-glycoprotein) restores sensitivity to multidrug-resistant cancer cells. Cancer Research.

[29] Allen JD, Schinkel AH (2002). Multidrug resistance and pharmacological protection mediated by the breast cancer resistance protein (BCRP/ABCG2). Molecular Cancer Therapeutics.

[30] Bates SE, Robey R, Miyake K, Rao K, Ross DD, Litman T (2001). The role of half-transporters in multidrug resistance. Journal of Bioenergetics and Biomembranes.

[31] Rabindran SK, Ross DD, Doyle LA, Yang W, Greenberger LM (2000). Fumitremorgin C reverses multidrug resistance in cells transfected with the breast cancer resistance protein. Cancer Research.

[32] Ahmed-Belkacem A, Pozza A, Macalou S, Perez-Victoria J, Boumendjel A, Pietro AD (2006). Inhibitors of cancer cell multidrug resistance mediated by breast cancer resistance protein (BCRP/ABCG2). Anti-Cancer Drugs.

[33] Imai Y, Tsukahara S, Ishikawa E, Tsuruo T, Sugimoto Y (2002). Estrone and 17β-Estradiol Reverse Breast Cancer Resistance Protein-mediated Multidrug Resistance. Japanese Journal of Cancer Research.

[34] Imai Y, Tsukahara S, Asada S, Sugimoto Y (2004). Phytoestrogens/flavonoids reverse breast cancer resistance protein/ABCG2-mediated multidrug resistance. Cancer Research.

[35] Mao Q, Unadkat J D (2015). Role of the breast cancer resistance protein (BCRP/ABCG2) in drug transport- an update. The AAPS journal.

[36] Liu P, Yu H, Sun Y, Zhu M, Duan Y (2012). A mPEG-PLGA-b-PLL copolymer carrier for adriamycin and siRNA delivery. Biomaterials.

[37] Maeda H, Bharate G, Daruwalla J (2009). Polymeric drugs for efficient tumor-targeted drug delivery based on EPR-effect. European Journal of Pharmaceutics And Biopharmaceutics.

[38] Torchilin V (2011). Tumor delivery of macromolecular drugs based on the EPR effect. Advanced Drug Delivery Reviews.

[39] Deshmukh M, Kutscher HL, Gao D, Sunil VR, Malaviya R, Vayas K, Stein S, Laskin JD, Laskin DL, Sinko PJ (2012). Biodistribution and renal clearance of biocompatible lung targeted poly (ethylene glycol) (PEG) nanogel aggregates. Journal of Controlled Release.

[40] Wang Y, Lu L, Shi J, Wang H, Xiao Z, Huang N (2011). Introducing RGD peptides on PHBV films through PEG-containing cross-linkers to improve the biocompatibility. Biomacromolecules.

[41] Bazile D, Prudhomme C, Bassoullet Mt, Marlard M, Spenlehauer G, Veillard M (1995). Stealth ME. PEG-PLA Nanoparticles Avoid Uptake By The Mononuclear Phagocytes System. Journal of Pharmaceutical Sciences.

[42] Zhou LP, Wu GY, Wei HJ, Guo ZY, Yang HQ, He YH, Xie SS, Liu Y (2014). Influence of different sized nanoparticles combined with ultrasound on the optical properties of in vitro normal and cancerous human lung tissue studied with OCT and diffuse reflectance spectra. Laser Physics.

[43] Wang TY, Choe JW, Pu KY, Devulapally R, Bachawal S, Machtaler S, Chowdhury SM, Luong R, Tian L, Khuri-Yakub B, Rao JH, Paulmurugan R, Willmann JK, Rao JH, Paulmurugan R, Willmann JK (2015). Ultrasound-guided delivery of microRNA loaded nanoparticles into cancer. Journal of Controlled Release.

[44] Eggen S, Fagerland SM, Morch Y, Hansen R, Sovik K, Berg S, Furu H, Bohn AD, Lilledahl MB, Angelsen A, Angelsen B, Davies CD (2014). Ultrasound-enhanced drug delivery in prostate cancer xenografts by nanoparticles stabilizing microbubbles. Journal of Controlled Release.

[45] Sadzuka Y, Nakai S, Miyagishima A, Nozawa Y, Hirota S (1997). Effects of administered route on tissue distribution and antitumor activity of polyethyleneglycol-coated liposomes containing adriamycin. Cancer Letters.

