# Supplementary material for: Enhanced therapeutic effect of Adriamycin on multidrug resistant breast cancer by the ABCG2-siRNA loaded polymeric nanoparticles assisted with ultrasound
Source: Oncotarget. 2015 Nov 9;6(41):43779–90. doi: 10.18632/oncotarget.6085 (PMC4791266; doi:10.18632/oncotarget.6085)
Supplement: Supplementary file 1 [file oncotarget-06-43779-s001.pdf]

SUPPLEMENTARY SCHEME FIGURE

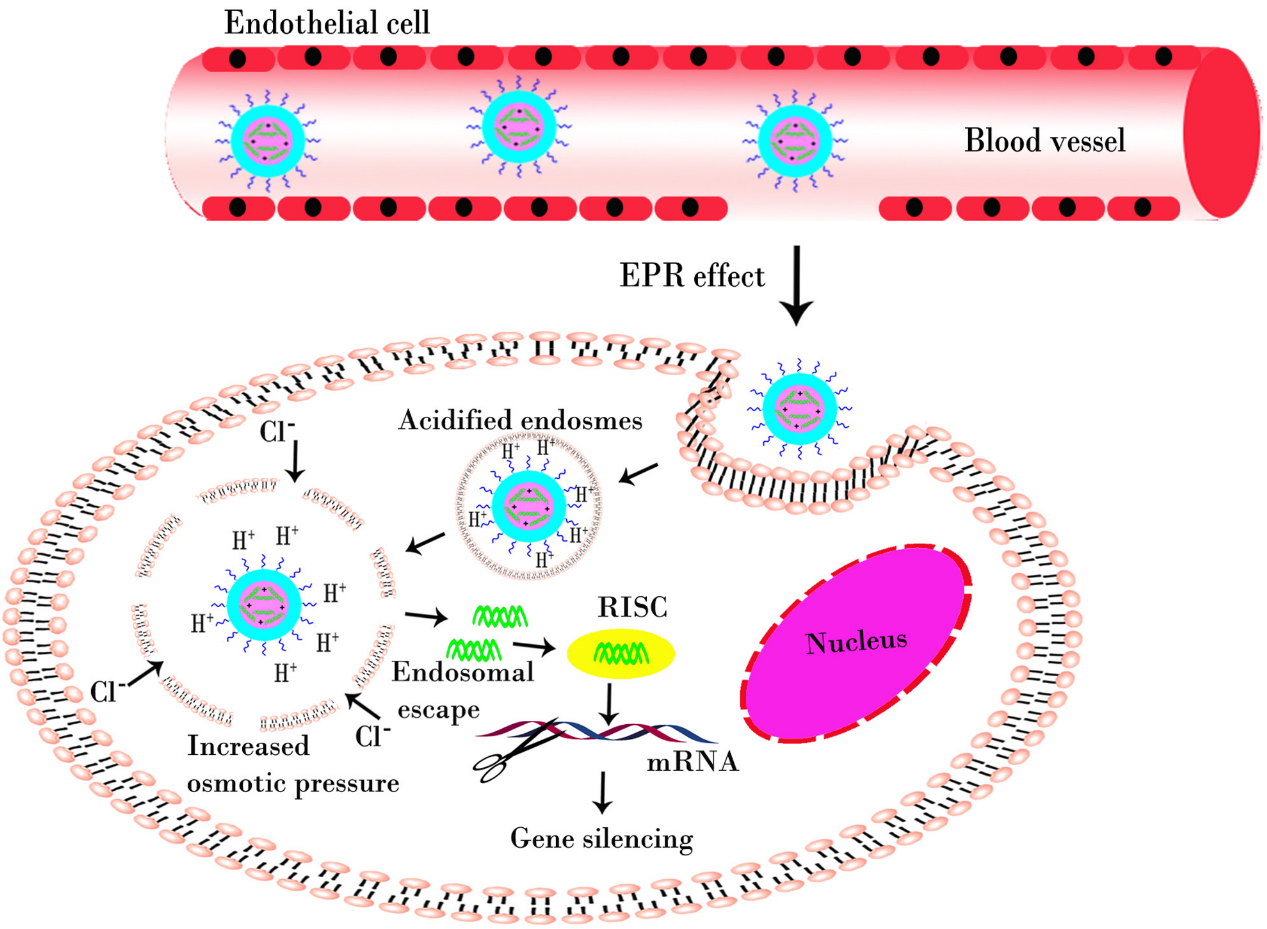

Supplementary Scheme Figure S1: Schematic illustration of the cellular uptake and intracellular trafficking pathway of ABCG2-siRNA-loaded PEAL NPs.
